# Liegetrauma: retrospektive Analyse einer Patientenkohorte aus einer universitären Notaufnahme

**DOI:** 10.1007/s00063-022-00912-w

**Published:** 2022-04-11

**Authors:** Christoph Hüser, Matthias Hackl, Victor Suárez, Ingo Gräff, Michael Bernhard, Volker Burst, Christoph Adler

**Affiliations:** 1 Klinik II für Innere Medizin: Nephrologie, Rheumatologie, Diabetologie und Allgemeine Innere Medizin, Medizinische Fakultät und Uniklinik Köln, Universität zu Köln, Köln, Deutschland; 2 Zentrale Notaufnahme, Medizinische Fakultät und Uniklinik Köln, Universität zu Köln, Köln, Deutschland; 3 Interdisziplinäres Notfallzentrum, Universitätsklinikum Bonn, Universität Bonn, Bonn, Deutschland; 4 Zentrale Notaufnahme, Universitätsklinikum Düsseldorf, Heinrich-Heine-Universität, Düsseldorf, Deutschland; 5 Klinik für Kardiologie, Angiologie, Pneumologie und internistische Intensivmedizin, Klinik III für Innere Medizin, Herzzentrum, Medizinische Fakultät und Uniklinik Köln, Universität zu Köln, Köln, Deutschland; 6 Institut für Schutz und Rettung, Berufsfeuerwehr Köln, Köln, Deutschland; 7 Klinik für Kardiologie, Angiologie, Pneumologie und internistische Intensivmedizin, Uniklinik Köln, Kerpener Str. 62, 50937 Köln, Deutschland

**Keywords:** Notfallmedizin, Nichttraumatologischer Schockraum, Immobilisation, Rhabdomyolyse, Liegend vorgefunden, Emergency medicine, Non-traumatic resuscitation room, Immobilization, Rhabdomyolysis, Found Down

## Abstract

**Hintergrund:**

Bisher fehlen Versorgungsdaten für Patienten mit Liegetrauma (LT).

**Methode:**

Deskriptive retrospektive Analyse aller rettungsdienstlich mit einem LT der Notaufnahme des Universitätsklinikums Köln von 07.2018 bis 12.2020 zugeführten Patienten.

**Ergebnis:**

Insgesamt konnten 50 Patienten mit LT (Altersmedian 76 Jahre, Liegedauer im Median 13,5 h) im Untersuchungszeitraum identifiziert werden. Die zugrunde liegende Ursache für das LT war in 40 % primär neurologisch (ischämischer Schlaganfall: 20 %, intrakranielle Blutung: 16 %, Epilepsie: 4 %), in 12 % eine Intoxikation und in 10 % ein häusliches Trauma. Häufige assoziierte Diagnosen waren Infektionen (52 %), Traumafolgen (22 %), Exsikkose (66 %), akute Nierenfunktionsstörung (20 %), schwere Rhabdomyolyse (Kreatininkinase ≥ 5000 U/l, 21 %) und schwere Hypothermie < 32 °C (20 %). Insgesamt wurden 69 % der Patienten auf einer Intensivstation aufgenommen und die Krankenhausletalität betrug 50 %.

**Schlussfolgerung:**

Das LT beschreibt einen Patientenzustand, bei dem infolge vielfältiger Ursachen plötzlich die eigenständige Mobilisierung und ein selbstständiges Hilfeholen verhindert werden und dadurch weitere Gesundheitsschäden entstehen. Bei diesem Syndrom sind Gewebsschäden als Folge des Liegens keine notwendige Voraussetzung für das Vorliegen eines LT. Aufgrund der hohen Morbidität und Letalität sollten diese Patienten in einem nichttraumatologischen Schockraum aufgenommen werden.

## Abstract (en)

**Background:**

Patients discovered recumbent, helpless and incapacitated, awake or unresponsive are referred to as “long lie trauma” (LLT) in the German medical jargon. Yet, a characterization of this cohort is missing.

**Methods:**

We retrospectively analyzed all LLT patients admitted to the emergency department of the University Hospital Cologne from July 2018 to December 2020.

**Results:**

A total of 50 LLT patients (median age 76 years, median time on the ground 13.5 h) were identified. The FD was most often attributed to primary cerebral causes in 40% of the cases (20% ischemic stroke, 16% intracranial hemorrhage, 4% epilepsy), intoxication/overdose (12%), and trauma (10%). It was often associated with infection (52%), injury (22%), hypovolemia (66%), acute kidney injury (20%), and severe rhabdomyolysis (creatine kinase ≥ 5000 U/l, 21%) as well as severe hypothermia < 32 °C (20%). Overall, 69% of the patients were admitted to an intensive care unit and in-hospital mortality was 50%.

**Conclusion:**

The term “long lie trauma” describes a complex clinical situation, in which various conditions lead to an incapacitated state with acute onset, which then causes further adverse health effects. Trauma or tissue damage were no obligatory requirement in this syndrome. Considering the high morbidity and in-hospital mortality, patients should initially be treated in the emergency room by an interdisciplinary team.

## Hintergrund

Obwohl der Begriff des *Liegetraumas* (LT) im prähospitalen und klinischen Kontext regelhaft verwendet wird, existiert bisher keine einheitliche Beschreibung dieses Patientenzustands. Dementsprechend finden sich in der Literatur gegenwärtig auch keine belastbaren Daten hinsichtlich Komplikationen und Letalität des LT. In den wenigen publizierten Kasuistiken wird unter einem LT beispielsweise ein gluteales Kompartmentsyndrom durch 10-stündiges Liegen nach Opiatabusus [6] oder eine Gesichtsschwellung – verursacht durch längeres Liegen mit herabhängendem Kopf – beschrieben [7]. Auch wenn eine Definition fehlt, scheint unter einem LT vor allem eine durch längeres Liegen ausgelöste muskuläre Minderperfusion mit konsekutiver Gewebsschädigung verstanden zu werden, die zu einer Rhabdomyolyse führt [14, 16]. Soweit den Autoren bekannt ist bis dato keine detaillierte Analyse und Charakterisierung einer Patientenkohorte mit LT im Kontext der klinischen Notfallversorgung in einer Notaufnahme erfolgt, obwohl in der englischen Literatur langes Liegen nach Stürzen bei älteren Patienten immer wieder als Risikofaktor für ein schlechtes Outcome diskutiert wird [2]. Ziel der vorliegenden Arbeit war es daher, aus einer Patientenkohorte mit LT die Einflussfaktoren auf Morbidität und Letalität zu identifizieren. Zudem sollten assoziierte Erkrankungen und die in der klinischen Versorgung benötigten Ressourcen erfasst und analysiert werden.

## Patienten und Methoden

Im Rahmen der retrospektiven monozentrischen Observationsstudie wurden Patienten, die im Zeitraum zwischen Juli 2018 und Dezember 2020 mit dem Stichwort „Liegetrauma“ durch den Rettungsdienst in der zentralen Notaufnahme (ZNA) der Universitätsklinik Köln zur Aufnahme kamen, konsekutiv eingeschlossen und analysiert. Da es sich beim LT um einen bisher undefinierten und nichtcodierbaren Begriff handelt, wurden Patienten anhand des Vorkommens des Stichworts „Liegetrauma“ in der Triagedokumentation der elektronischen Patientendokumentation identifiziert, um ein möglichst unverfälschtes Bild des im Alltag unter „Liegetrauma“ verstandenen Kollektives zu erhalten. Aus der Routinedokumentation der ZNA wurden folgende Parameter der identifizierten Patienten in einer elektronischen Datenbank erfasst: demographische Daten (Alter, Geschlecht), Auffindeort, Liegedauer bzw. das Zeitintervall zwischen letztem Kontakt mit dem sozialen Umfeld in Wohlbefinden und Auffinden mit LT („last seen well“), initiale Glasgow Coma Scale (GCS) beim Eintreffen des Rettungsdiensts. Aus den routinemäßig innerklinisch im Rahmen der notfallmedizinischen Versorgung in der ZNA dokumentierten Patientenverläufen wurden folgende Parameter eruiert: Vitalparameter (Herzfrequenz, Blutdruck), S_p_O_2_, tympanische Temperatur, GCS (soweit nicht intubiert und beatmet), der klinische Eindruck zum Volumenstatus, initiale Blutgasanalyse (BGA) und Laborparameter (aus dem Blut, soweit vorhanden auch Urin). Darüber hinaus wurden die dem LT zugrunde liegende Ursache, assoziierte Diagnosen und Begleitverletzungen aus der Patientenakte entnommen. Dabei kamen folgende Definitionen zur Anwendung: Eine hämodynamische Instabilität wurde definiert als Katecholaminpflichtigkeit oder Hypotonie mit einem systolischem Blutdruck < 90 mm Hg. Als Hypothermie wurde definiert eine tympanische Körpertemperatur < 35 °C, als eine schwere Hypothermie eine Körpertemperatur < 32 °C. Als Rhabdomyolyse wurde ein Anstieg der Kreatininkinase (CK) auf Werte von mindestens 1000 U/l gewertet, als ausgeprägte Rhabdomyolyse ein Anstieg der CK auf Werte von mindestens 5000 U/l [5]. Eine akute Nierenfunktionsstörung wurde entsprechend der KDIGO(„Kidney Disease: Improving Global Outcomes“)-Kriterien definiert [12]. Ein positives Ethikvotum der Ethikkommission der medizinischen Fakultät der Universität zu Köln lag vor (Zeichen 2021-17).

## Statistik

Diagnosen, Vital- und Laborparameter sowie relevante Untersuchungsbefunde der Patienten wurden anonymisiert erfasst. Es erfolgte die deskriptive statistische Auswertung mit Ausgabe von Mittelwert, Median, Minimum und Maximum und Standardabweichung. Bei Nominalskalierung erfolgte die Ausgabe als Häufigkeit (%). Fehlende Werte wurden nicht ersetzt und nicht in die Auswertung mit eingezogen. Vergleiche zwischen den Gruppen erfolgten für die dichotom verteilten Daten mittels Fishers exaktem Test, Korrelationen wurden mittels Pearson-Test bestimmt. Als statistisch signifikant wurde ein *p*-Wert < 0,05 angesehen. Die Auswertung erfolgte mittels IBM SPSS Statistics 27 (Armonk, NY, USA).

## Ergebnisse

Insgesamt wurden in dem untersuchten Zeitraum 50 Patienten mit LT identifiziert. Alle Patienten wurden durch den Rettungsdienst im häuslichen Umfeld unfähig, eigenständig Hilfe zu holen, vorgefunden. Die Patienten (männlich: 58 %) waren im Median 76 Jahre alt (Mittelwert: 74 ± 14 Jahre, Minimum – Maximum (Min–Max): 36–103 Jahre). Nur 14 % der Patienten waren jünger als 60 Jahre.

### Liegedauer und präklinische GCS

Die exakte Liegedauer konnte bei 10 Patienten (20 %) ermittelt werden und betrug im Median 13,5 h (Mittelwert: 13,5 h [23 ± 26 h], Min–Max: 9–96 h). Das Last-seen-well*-*Intervall lag im Median bei 24 h (Mittelwert: 24 h [40 ± 28 h], Min–Max: 7–96 h) und konnte bei 27 Patienten (54 %) erhoben werden. Die initiale präklinische mittlere GCS lag bei 9 ± 5.

### Aufnahmezustand

Bei Übergabe in der ZNA waren 48 % der Patienten endotracheal intubiert und maschinell beatmet. Es wurden 29 Patienten der Triagekategorie 1 nach Manchester Triage System zugeordnet, 12 Patienten der Kategorie 2, 5 Patienten der Kategorie 3 und 2 Patienten der Kategorie 4. Bei einem Patienten war die Triagekategorie nicht dokumentiert. Der Großteil der Patienten zeigte abgesehen von der GCS stabile Vitalparameter und nur 10 % der Patienten waren hämodynamisch instabil. Zum Zeitpunkt der Vorstellung waren mehr als zwei Drittel des Kollektivs dem klinischen Eindruck nach exsikkiert und 23 % der Patienten hypotherm, wobei 20 % der Patienten eine schwere Hypothermie zeigten. Die detaillierten Charakteristika der Studienpopulation sind in Tab. 1 zusammengefasst.

**Table 1:** 

*Alter (Jahre), n* *=* *50*	*Median (Mittelwert* *±* *SD), Min–Max*
*Gesamt*	76 (74 ± 14), 36–103
Weiblich (21, 42 %)	81 (77 ± 15), 38–103
Männlich (29, 58 %)	74 (71 ± 13), 36–92
*Liegedauer (h), n* *=* *10*	13,5 (23 ± 26), 9–96
*„Last seen well“ (h), n* *=* *27*	24 (40 ± 28), 7–96
*Patientin intubiert durch Rettungsdienst, n* *=* *42*	*n (%)*
Ja	20 (48 %)
*Initiale Glasgow Come Scale (GCS) bei Eintreffen des Rettungsdiensts, n* *=* *45*	*Median (Mittelwert* *±* *SD), Min–Max*
–	8 (9 ± 5), 3–15
–	*n* (%)
GCS < 9	25 (56 %)
GCS 3 oder 4	17 (38 %)
GCS 5 oder 6	4 (9 %)
GCS 7 oder 8	4 (9 %)
GCS 9 oder 10	1 (2 %)
GCS 11 oder 12	1 (2 %)
GCS 13	1 (2 %)
GCS 14 oder 15	17 (38 %)
Herzfrequenz (/min), *n* = 44	Median (Mittelwert ± SD)
–	89 (91 ± 31)
–	*n* (%)
< 50/min	2 (5 %)
Bradykard (< 60/min)	6 (14 %)
Tachykard (> 100/min)	14 (30 %)
> 150/min	3 (7 %)
*Systolischer Blutdruck (RR*_*sys*_*) bei Vorstellung (mm* *Hg), n* *=* *47*	*Median (Mittelwert* *±* *SD)*
–	140 (135 ± 34)
–	*n* (%)
RR_sys_ < 90 mm Hg	4 (9 %)
RR_sys_ > 140 mm Hg	21 (45 %)
RR_sys_ > 160 mm Hg	8 (17 %)
*Diastolischer Blutdruck (RR*_*dias*_*) bei Vorstellung (mm* *Hg), n* *=* *47*	*Median (Mittelwert* *±* *SD)*
–	80 (78 ± 20)
*Periphere Sauerstoffsättigung (S*_*p*_*O*_*2*_*) bei Vorstellung (%), n* *=* *45*	*Median (Mittelwert* *±* *SD)*
Mittelwert	98 (96 ± 9)
–	*n (%)*
S_p_O_2_ < 90 %	4 (9 %)
S_p_O_2_ < 80 %	3 (7 %)
*Temperatur (°C), n* *=* *30)*	*Median (Mittelwert* *±* *SD), Min–Max*
Mittelwert	36,2 (34,8 ± 3,5), 24,3–38,3
–	*n (%)*
Temperatur mind. 38 °C	1 (3 %)
Temperatur < 35 °C	7 (23 %)
Temperatur < 32 °C	6 (20 %)
Temperatur < 28 °C	2 (7 %)
*Klinischer Eindruck Volumenstatus, n* *=* *38*	*n (%)*
Exsikkiert	25 (66 %)
Euvolämisch	12 (32 %)
Hypervolämisch	1 (3 %)

## Laborwerte bei Aufnahme

Laborparameter zum Aufnahmezeitpunkt lagen – soweit nicht anders berichtet – bei 48 der 50 eingeschlossenen Patienten (96 %) vor.

### Rhabdomyolyse

Bei der Vorstellung wurde bei 85 % der Patienten eine erhöhte CK nachgewiesen (≥ 190 U/l). Dabei lag die initiale CK zum Zeitpunkt der ersten Blutabnahme bei 58 % der Patienten bei ≥ 1000 U/l und bei 21 % der Patienten bei ≥ 5000 U/l. Eine CK > 100.000 U/l wies nur ein Patient auf. Die maximale CK lag bei 72 % der Patienten am Aufnahmetag, bei 16 % am Tag nach der Aufnahme und bei 9 % der Patienten am zweiten Tag nach der Aufnahme vor.

### Nieren- und Elektrolytstörungen

Bei der Hälfte der Patienten wurde bei Aufnahme ein über dem Normwert von 1,1 mg/dl liegendes Kreatinin nachgewiesen, bei 21 % lag dieses > 2 mg/dl. Schwere Entgleisungen der Serumelektrolyte waren selten: 8 % der Patienten zeigten eine Hyponatriämie < 130 mmol/l, wobei der minimale Natriumwert bei 122 mmol/l lag. Eine Hyperkaliämie mit einem Serumkalium von mindestens 6 mmol/l wiesen 8 % der Patienten auf.

### Entzündungswerte

Ein erhöhtes CRP von mindestens 5 mg/l zeigte sich im initialen Labor bei 76 % der Patienten. Eine Leukozytose (Leukozyten > 11,3 × 10^9^/ml) fand sich bei 73 % der Patienten und eine Leukopenie (Leukozyten < 4,4 × 10^9^/ml) bei 2 % der Patienten.

### Blutgasanalyse (BGA)

Hier lagen Werte einer venösen BGA bei Aufnahme für 47 Patienten vor. Initial zeigten 57 % der Patienten ein Laktat > 2,2 mmol/l. Bei 28 % der Patienten lag dies ≥ 4 mmol/l und bei 13 % ≥ 10 mmol/l. Der initiale pH-Wert lag bei 49 % der Patienten unter 7,35 und bei 15 % unter 7,2. Bei 9 % der Patienten war der initiale pH-Wert > 7,45. Die häufigste Störung des Säure-Basen-Haushalts war eine metabolische Acidose oder eine kombiniert metabolisch-respiratorische Acidose bei 48 % der Patienten. Der mittlere Blutzucker lag bei 159 mg/dl (± 82), wobei der minimale Blutzucker bei 66 mg/dl und der maximale Blutzucker bei 495 mg/dl lag.

## Ursachen des Liegetraumas und assoziierte Diagnosen

Ausgehend von den Krankenhausentlassdiagnosen wurden 40 % der Fälle mit LT auf eine neurologische Ursache zurückgeführt. In 12 % wiesen Patienten mit LT Intoxikationen als ursächliches Ereignis auf. Ein primäres Trauma zählte mit 10 % zu den weiteren Ursachen eines LT, wobei in 2 Fällen ein traumatischer Querschnitt und in 3 Fällen eine primär traumatische intrakranielle Blutung vorlag. In 8 % der Fälle wurde das LT auf eine Infektion zurückgeführt. Einzelne Fälle wurden diversen Ursachen (u. a. Hyponatriämie, „frailty“, Hypoglykämie) zugeschrieben (Tab. 2).

**Table 2:** 

*Ursache Liegetrauma, n* *=* *50*	*n (%)*
Ischämischer „stroke“	10 (20 %)
Unklar	10 (20 %)
Nichttraumatische intrakranielle Blutung (ICB)	8 (16 %)
Intoxikation	6 (12 %)
Trauma (inkl. traumatischer ICB)	5 (10 %)
Sonstiges	3 (6 %)
Epilepsie	2 (4 %)
Sepsis	2 (4 %)
Meningitis	2 (4 %)
Gastrointestinale Blutung	2 (4 %)
*Assoziierte Diagnosen, n* *=* *50*	*n (%)*
Infektion	26 (52 %)
Davon Pneumonie	18 (69 %)
Davon Harnwegsinfekt	6 (23 %)
Traumafolge mit unfallchirurgischer Versorgung	11 (22 %)
Akutes Nierenversagen	10 (20 %)
Sturzereignis	9 (18 %)
Substanzabusus vorbekannt	9 (18 %)

Neben den als ursächlich gesehenen Diagnosen fanden sich weitere häufig mit dem LT assoziierte Diagnosen. Bei insgesamt 52 % der Patienten (inkl. der 8 % Patienten mit als ursächlich für das Liegetrauma gewerteter Infektion) lag eine Infektion vor, diese war zu 69 % eine Pneumonie (meist auf Aspiration zurückgeführt) und zu 23 % ein Harnwegsinfekt. Bei jedem 5. Patienten wurde eine akute Nierenfunktionsstörung diagnostiziert. Im Rahmen des stationären Aufenthalts war eine Dialysebehandlung bei 11 % der Patienten indiziert, es war kein Patient vorbestehend dialysepflichtig. Inklusive der als Ursache des Liegetraumas gewerteten Traumata benötigten 22 % der Patienten eine unfallchirurgische Versorgung, wobei Sturzfolgen, wie Brüche und Schnitt- sowie Platzwunden, hier typische Indikationen waren. Bei 18 % der Patienten war ein Substanzabusus vorbekannt.

## Disposition und Behandlungsergebnis

Aus der ZNA mussten 69 % der Patienten einer intensivmedizinischen Versorgung zugeführt werden. Ein Patient verstarb unmittelbar in der Notaufnahme. Insgesamt verstarb die Hälfte des analysierten Patientenkollektivs im Rahmen des Krankenhausaufenthalts. Dabei zeigte sich im Trend bei Patientin mit initial niedrigerer GCS eine höhere Letalität, ebenso war die Letalität von Patienten mit einem Last-seen-well-Intervall von mindestens 24 h höher als bei Patienten mit kürzerem Intervall (Tab. 3). Hingegen wies weder die als ursächlich gewertete Diagnose noch die Höhe der initialen CK eine Korrelation mit der Krankenhausletalität auf. Von den Patienten, die nicht im Krankenhausaufenthalt verstarben, konnten 48 % nach einer Aufenthaltsdauer von im Mittel 14 ± 8 Tagen (Median: 16 Tage) in das häusliche Umfeld entlassen werden, 17 % wurden in eine Akutgeriatrie verlegt und 22 % einer neurologischen Frührehabilitation oder sonstigen Rehabilitationsmaßnahme zugeführt.

**Table 3:** 

	Verstorben im Krankenhaus, *n* (%)
*Initiale GCS RD*	
GCS 3 oder 4 (*n* = 17)	12 (71 %)
GCS 5–8 (*n* = 7)	4 (57 %)
GCS 9–13 (*n* = 3)	1 (33 %)
GCS 14 oder 15 (*n* = 14)	5 (36 %)
*„Last seen well“*	
7–< 24 h (*n* = 7)	2 (29 %)
24–< 48 h (*n* = 8)	5 (63 %)
48–< 72 h (*n* = 4)	3 (75 %)
72 h und mehr (*n* = 6)	4 (67 %)
*Intensivverlegung*	
Verlegung auf IMC/ITS (*n* = 33)	20 (61 %)
Keine Verlegung auf IMC/ITS (*n* = 13)	3 (23 %)
*Ursache Liegetrauma*	
ICB (*n* = 8)	7 (88 %)
Ischämischer „stroke“ (*n* = 10)	7 (70 %)
Epilepsie (*n* = 2)	1 (50 %)
Gastrointestinale Blutung (*n* = 2)	1 (50 %)
Meningitis (*n* = 2)	1 (50 %)
Sepsis (*n* = 2)	1 (50 %)
Unklar (*n* = 6)	3 (50 %)
Trauma (*n* = 5)	2 (40 %)
Sonstiges (*n* = 3)	0 (0 %)
Intoxikation (*n* = 6)	0 (0 %)
*Initiale CK*	
CK < 1000 U/l (*n* = 18)	10 (56 %)
CK > 1000 < 5000 U/l (*n* = 16)	6 (38 %)
CK > 5000 U/l (*n* = 10)	5 (50 %)

## Diskussion

Die vorliegende retrospektive Untersuchung zeigt erstmals die wesentlichen Ursachen, Häufigkeiten assoziierter Erkrankungen und den innerklinischen Verlauf für Patienten mit LT und weist eine mit 50 % hohe Letalität für dieses Patientenkollektiv nach. Patienten mit LT waren kritisch krank, mussten häufig endotracheal intubiert und beatmet werden und wurden in 69 % der Fälle auf eine Intensivstation aufgenommen.

In dem retrospektiv untersuchten Kollektiv von Patienten mit LT, die sich in einer ZNA vorstellten, wurden als häufige Ursachen eine neurologische Pathologie, eine Intoxikation und ein initiales Trauma identifiziert. Dabei traten bei Patienten mit LT folgende Erkrankungen in einem hohen Prozentsatz auf: Hypothermie, Exsikkose, Nierenversagen, Störungen des Säure-Basen-Haushalts und Infektionen. Eine ausgeprägte Rhabdomyolyse (CK ≥ 5000 U/l) wurde bei Aufnahme nur bei 21 % der Patienten beobachtet und auch die maximale CK lag nur bei 26 % ≥ 5000 U/l. Eine schwere muskuläre Verletzung oder andere Gewebsschädigung, die durch das LT ausgelöst wurden, schienen in dieser Kohorte nicht pathognomonisch vorzuliegen, was in einem gewissen Kontrast zu den bisher veröffentlichten Publikationen steht [6, 7]. Gleichzeitig stand die Gewebsschädigung und Rhabdomyolyse in unserer Untersuchung ebenso wie in anderen Untersuchungen nicht im Vordergrund des Patientenzustands [14]. Vielmehr scheint die hohe Krankenhausletalität in unserer Kohorte maßgeblich in einer Kombination aus ursächlicher Erkrankung und Liegedauer begründet zu sein, wobei im Trend neurologische Diagnosen und ein Last-seen-well-Intervall von mindestens 24 h mit einer höheren Sterblichkeit einhergingen. So korrelierte auch in einer populationsbasierten prospektiven Untersuchung aller vom Rettungsdienst San Francisco über 12 Wochen tot oder hilflos in ihrer Wohnung vorgefundenen Patienten die Sterblichkeit der lebend aufgefundenen Patienten statistisch signifikant mit der Liegedauer [9]. In dieser Studie waren jedoch allgemeine Schwäche und Stürze in mehr als der Hälfte der Fälle für die Immobilisation verantwortlich, während ein Koma nur bei 2 % der Patienten vorlag. Im Gegensatz zu unserem Kollektiv verstarben in dieser US-amerikanischen Studie nur 10 % der stationär behandlungsbedürftigen Patienten, wobei immerhin auch 52 % dieser Patienten auf eine Intensivstation aufgenommen werden mussten. Hingegen zeigt eine retrospektive monozentrische Analyse von 854 Patienten, die in der Berliner Charité mit bei Vorstellung in der Notaufnahme persistierendem unklarem Koma behandelt wurden, eine im Vergleich mit unserer Untersuchung ähnliche Häufigkeit an primär zerebralen Läsionen (22 % ICB, 11 % ischämischer „stroke“) und Intoxikationen (19 %), jedoch viel häufiger eine Epilepsie (22 %) als Ursache des Komas [15]. Obwohl hier nur Patienten mit persistierendem Koma untersucht wurden und unsere Kohorte auch etwa ein Drittel Patienten ohne Bewusstseinsstörungen umfasst, lag die Krankheitsletalität der Berliner Studie mit 25 % ebenfalls deutlich unterhalb der unserer Kohorte. In unserem Kollektiv lag die Letalität bei 50 % und war somit auch auffallend höher als in den deutschen OBSERvE-Studien zu Patienten, die eine nichttraumatologische Schockraumversorgung benötigten (34–36 %; [8]). Dieser Vergleich unterstreicht, dass die hohe Krankenhausletalität in unserer Kohorte nicht nur auf die Schwere der zugrunde liegenden Erkrankungen, sondern vermeintlich auch auf die zusätzliche Liegedauer mit ihren Komplikationen zurückzuführen ist.

Dabei bleibt der Vergleich mit anderen Untersuchungen erschwert, da keine deutsche Fallserie sich bisher mit Patienten mit LT befasst hat, im Englischen kein Pendant zum Begriff LT zu bestehen scheint und einheitliche Definitionen zur Schweregraderfassung nichttraumatologischer Patienten fehlen. Im angloamerikanischen Raum ist dem LT am nächsten kommend noch der unspezifische Terminus „found down“ verbreitet [10, 11, 13]. Hierunter werden jedoch hilflos, aber bewusstseinsgetrübt vorgefundene Patienten verstanden, die (beispielshaft mit Zahlen von Howard et al. [9]) einen hohen Anteil an Intoxikationen mit Alkohol (33 %) und anderen Substanzen aufweisen (19 %) sowie häufig obdachlos sind (25 %). Somit beschreibt diese Begrifflichkeit ein deutlich anderes Patientenkollektiv als das unsere. Ein weiterer ähnlicher Begriff im Englischen bezeichnet das „long lie“, worunter eine Liegedauer von mehr als einer Stunde nach Stürzen vor allem bei älteren Patienten verstanden wird [4]. Die Unfähigkeit des eigenständigen Aufstehens ist dabei als Risikofaktor für eine erhöhte Mortalität auch in einer Metaanalyse bestätigt worden [2]. In einer beispielhaften monozentrischen und prospektiven Beobachtungsstudie aus dem Jahr 2006 von 417 Patienten im Alter von mindestens 75 Jahren, die nach einem Sturzereignis in einer Notaufnahme vorstellig wurden, wurden die meisten Stürze durch Unfälle ausgelöst (65 %), während auslösende Erkrankungen seltener waren. Es wurden ähnliche mit dem langen Liegen assoziierte Diagnosen wie bei uns gefunden, wobei Trauma bei 83 % der Patienten eine deutlich häufigere Konsequenz war und metabolische oder medizinische Störungen, wie Rhabdomyolyse, Hypothermie und Säure-Base-Störungen, nur bei 22 % der Patienten auftraten. Auch die Sterblichkeit war mit einer 6‑Monats-Letalität von 15 % insgesamt niedriger.

Die hohe Sterblichkeit und Morbidität in unserer Kohorte von Patienten mit LT, die im Vergleich mit der Literatur auf einer Kombination aus der Schwere der auslösenden Erkrankung und den Komplikationen des langen Liegens zu beruhen scheint, rechtfertigt aus unserer Sicht, grundsätzlich bei Ankündigung eines Patienten mit LT die – zum Teil auch schon geforderte [1] – Indikation zur Schockraumversorgung zu stellen, um den Patienten die adäquate Versorgung zukommen lassen zu können [3]. Ob diese Folgerung unserer Pilotstudie sich reproduzieren und generalisieren lässt, sollte eine größere multizentrische Untersuchung klären.

## Limitationen

Eingeschränkt wird die Aussagekraft unserer Studie durch die kleine Zahl an untersuchten Patienten, bei denen aufgrund des retrospektiven Studiencharakters und der schlechten Dokumentationsqualität in der Notfallversorgung zum Teil eine hohe Quote an fehlenden Werten vorlag. Unterschiede zwischen einzelnen Subgruppen ließen sich somit nur schwer evaluieren und meist nur als Trend erkennen. Ob diese nur zufällig oder auch statistisch signifikant sind, ließe sich erst mit einer größeren Patientenkohorte klären. Eine weitere Limitation besteht darin, dass nur Patienten mit dem Stichwort „Liegetrauma“ in der Triagedokumentation eingeschlossen wurden, um keine Verzerrung des bisher nicht definierten Begriffs durch eine vorherige Definition zu erzeugen. Dadurch konnte jedoch wahrscheinlich nicht jeder Patient mit LT im Untersuchungszeitraum identifiziert werden. Die so identifizierten Patienten konnten jedoch ohne Kenntnis der späteren Diagnosen eingeschlossen werden und so ein möglichst unverzerrtes Bild des Patientenkollektivs mit LT bieten.

Eine maßgebliche weitere Limitation dieser hypothesengenerierenden Pilotstudie ist, dass sie monozentrisch an einem Maximalversorger durchgeführt wurde und hierdurch ein deutlicher Bias der Daten entstehen kann, weil der Rettungsdienst möglicherweise schwerer erkrankte oder selektierte Patienten eingeliefert hat. Die im Vergleich zu anderen Studien sehr hohe Krankenhausletalität könnte als Hinweis hierfür gewertet werden. Andererseits bestand zumindest die Charité-Kohorte von Schmidt et al. ebenfalls aus schwerkranken, nämlich komatösen, Patienten eines Maximalversorgers und hier war die Letalität im Gegensatz zu unseren Patienten deutlich geringer.

## Fazit für die Praxis

- Das Liegetrauma (LT) ist ein komplexes klinisches Bild, bei dem durch vielfältige Ursachen plötzlich die eigenständige Mobilisierung und ein Hilfeholen verhindert werden und dadurch weitere Gesundheitsschäden entstehen.
- Ursächlich waren häufig neurologische Erkrankungen, gefolgt von Intoxikationen und Trauma für das LT verantwortlich.
- Patienten mit LT zeigten in dieser limitierten Kohorte über die verschiedenen Ursachen hinweg eine hohe Morbidität und Letalität, die eine nichttraumatologische Schockraumversorgung indizieren.
- Im Rahmen der innerklinischen Versorgung muss aktiv nach Infektionen, Hypothermie, Exsikkose, Nierenfunktions- und Säure-Basen-Störungen sowie Verletzungen/Muskelschäden gefahndet und diese therapiert werden.

## References

[1] Bernhard M, Ramshorn-Zimmer A, Hartwig T (2014). Schockraummanagement kritisch erkrankter Patienten. Anders als beim Trauma? (Management of critically ill patients in the resuscitation room. Different than for trauma?). Anaesthesist.

[2] Bloch F (2012). Critical falls. Why remaining on the ground after a fall can be dangerous, whatever the fall. J Am Geriatr Soc.

[3] Bogner-Flatz V, Hinzmann D, Kanz K-G (2020). Der Schockraum als Nahtstelle zwischen Präklinik und Klinik. Notarzt.

[4] Fleming J, Brayne C (2008). Inability to get up after falling, subsequent time on floor, and summoning help. Prospective cohort study in people over 90. BMJ.

[5] Gaik CWT (2020). Rhabdomyolyse. Ein Überblick zu Pathophysiologie, Diagnostik und Therapie. Anasth Intensivmed.

[6] Gleich J, Fürmetz J, Kamla C (2020). Gluteales Kompartmentsyndrom nach Liegetrauma bei Opiatabusus (Gluteal compartment syndrome after immobilization following opioid abuse). Unfallchirurg.

[7] Gräff I, Dolscheid-Pommerich RC, Ghamari S (2018). Verwahrlost, einsam und krank – der soziale Breakdown. Eine spezielle Patientengruppe in der zentralen Notaufnahme (Neglected, lonely and sick—the social breakdown : A special patient group in the emergency department). Med Klin Intensivmed Notfmed.

[8] Grahl C, Hartwig T, Weidhase L (2021). Früher innerklinischer Verlauf kritisch kranker nichttraumatischer Patienten im Schockraum einer deutschen Notaufnahme (OBSERvE2-Studie) (Early in-hospital course of critically ill nontrauma patients in a resuscitation room of a German emergency department (OBSERvE2 study)). Anaesthesist.

[9] Gurley RJ, Lum N, Sande M (1996). Persons found in their homes helpless or dead. N Engl J Med.

[10] Howard BM, Kornblith LZ, Conroy AS (2015). The found down patient. A western trauma association multicenter study. J Trauma Acute Care Surg.

[11] Jacobs BG, Turnipseed SD, Nguyen AN (2015). Acute medical diagnoses are common in “found down” adult patients presenting to the emergency department as trauma. J Emerg Med.

[12] Kidney Disease: Improving Global Outcomes (KDIGO) Acute Kidney Injury Work Group (2012). KDIGO clinical practice guideline for acute kidney injury. Kidney Int Suppl.

[13] Kornblith LZ, Kutcher ME, Evans AE (2013). The “found down” patient. A diagnostic dilemma. J Trauma Acute Care Surg.

[14] Ratcliffe PJ, Ledingham JG, Berman P (1984). Rhabdomyolysis in elderly people after collapse. BMJ.

[15] Schmidt WU, Ploner CJ, Lutz M (2019). Causes of brain dysfunction in acute coma. A cohort study of 1027 patients in the emergency department. Scand J Trauma Resusc Emerg Med.

[16] Wongrakpanich S, Kallis C, Prasad P (2018). The study of rhabdomyolysis in the elderly. An epidemiological study and single center experience. A&D.

